# Hallmarks of perineural invasion in pancreatic ductal adenocarcinoma: new biological dimensions

**DOI:** 10.3389/fonc.2024.1421067

**Published:** 2024-07-25

**Authors:** Yaquan Sun, Wei Jiang, Xiang Liao, Dongqing Wang

**Affiliations:** ^1^ Institute of Medical Imaging and Artificial Intelligence, Jiangsu University, Zhenjiang, China; ^2^ Department of Medical Imaging, The Affiliated Hospital of Jiangsu University, Zhenjiang, China

**Keywords:** pancreatic ductal adenocarcinoma, perineural invasion, tumor microenvironment, neural reprogramming, pain

## Abstract

Pancreatic ductal adenocarcinoma (PDAC) is an aggressive malignant tumor with a high metastatic potential. Perineural invasion (PNI) occurs in the early stages of PDAC with a high incidence rate and is directly associated with a poor prognosis. It involves close interaction among PDAC cells, nerves and the tumor microenvironment. In this review, we detailed discuss PNI-related pain, six specific steps of PNI, and treatment of PDAC with PNI and emphasize the importance of novel technologies for further investigation.

## 1 Introduction

Pancreatic ductal adenocarcinoma (PDAC) is the fourth-leading cause of cancer-related death in the United States of America, with a 5-year overall survival rate of 13% (1). In 2024, 66440 new PDAC cases and 51750 PDAC-related deaths were reported in the United States of America. Metastasis is the leading cause of PDAC-related death, and patients with metastatic PDAC have a 5-year survival rate of <8%. Local metastasis occurs more frequently than distant metastasis in PDAC (2).

Perineural invasion (PNI) is the most common type of local metastasis in PDAC, with a high incidence, a special invasion routine and life-threatening clinical manifestations. Patients with PNI have a poor quality of life and prognosis (**Table 1**) (3). PNI is defined as the presence of cancer cells along the pancreatic nerve and peripheral nerve plexus within the epineural, perineural and endoneurial spaces of the neuronal sheath, and independently meet the situation that cancer cells envelop at least 33% of the nerves (9). It may result in inflammation, pain, sensory abnormalities, numbness and paralysis (3). Treatment of PDAC with PNI is difficult, and the rate of local or regional recurrence is as high as 60% (10), which may be attributed to the presence of residual cancer cells in the nerve sheath. PNI occurs as a result of the interaction among cancer cells, nerve cells and the tumor microenvironment (TME). However, the precise mechanisms underlying PNI in PDAC remain unclear (11).

**Table 1:** Cancers and perineural invasion incidence rate.

Cancer type	PNI incidence rate(%)	Reference
Pancreatic cancer	~100	(3, 4)
Head and neck cancers	~80	(5)
Oral cancer	~33	(3)
Prostate cancer	~80	(6)
Stomach cancer	~60	(7)
Colorectal cancer	~30	(8)

In this review, we focus on the key steps of PNI and associated pain in PDAC. PNI is a continuous multistep process that involves various factors. The complex process of PNI is divided into six steps as follows: mutual chemotaxis between cancer and nerve cells, extracellular matrix (ECM) remodeling, cancer cell adhesion and invasion, immune evasion and nerve remodeling and regeneration. In addition to sumarising these steps, we discuss the recent research progress of PNI and novel strategies for targeting PNI in the treatment of PDAC.

## 2 Anatomical basis of PNI in PDAC and nerve physiological functions

The high incidence of PNI in PDAC is mainly attributed to the specific anatomical location of the pancreas and the unique nerve plexus surrounding the organ (12). As an extraperitoneal organ, the pancreas lies transversely in the upper abdomen between the duodenum on the right and the spleen on the left and is enriched with the nerve plexus. The pancreas can be divided into the head, neck, body and tail (13). According to the peripancreatic nerve division standard established by the Japan Pancreas Society (JPS), the peripancreatic nerve plexus is divided into six subgroups, namely, pancreas head nerve plexus I (PLX- I) and II (PLX-II), hepatoduodenal ligament-surrounding nerve plexus (PLX-hdl), superior mesenteric artery-surrounding nerve plexus (PLX-sma), right celiac ganglion plexus (Rcg) and left celiac ganglion plexus (Lcg). PLX- I extends from the celiac ganglion to the medial and superior uncinate processes, whereas PLX-II extends from the superior mesenteric artery nerve plexus to the medial and superior uncinate processes of the pancreas (14). Based on the subregions of the nerve plexus, PNI can occur through the following pathways (15): (1) PPC1 pathway, in which cancer cells in the pancreatic uncinate process (middle and upper regions) disseminate posterior to the portal vein to the right celiac ganglion; (2) PPC2 pathway, in which cancer cells in the pancreatic uncinate process (the tail region) travel along the inferior posterior pancreaticoduodenal artery or the jejunal trunk of the superior mesenteric vein to the mesenteric ganglion; PNI occurs through the PPC2 pathway in approximately 74%–81% of PDAC cases; (3) mesentery pathway, in which cancer cells in the pancreatic uncinate process disseminate posterior to the inferior pancreaticoduodenal artery to the mesentery of the small intestine or the transverse mesocolon; (4) anterior pathway, in which head and neck pancreatic cancer cells invade the right celiac ganglion through the gastroduodenal artery or the common hepatic artery. Moreover, pancreatic cancer cells may spread along the lymphatic capillaries and invade the superior mesenteric artery nerve plexus, eventually leading to PNI.

In addition to the specific anatomical location of the pancreas, the characteristic distribution of nerve fibers in the pancreas significantly contributes to PNI. Zuo et al. reported that nerve fibers surrounding the pancreas are rich in blood supply and form a network, facilitating the interaction between cancer cells and nerves (14). The pancreas is innervated by two types of peripheral nerves, the autonomic nerve and the sensory nerve (16). The sympathetic and parasympathetic nerves form the autonomic nervous system. The sympathetic nervous system is distributed in areas such as the pancreatic ganglia, vascular system, endocrine islands, ducts, and lymph nodes (16). The function of the parasympathetic nervous system is related to the secretion of insulin. Mainly by increasing the release of digestive enzymes to reduce insulin secretion. In terms of sensory nerves, different functional nerve fibers are distributed in various parts of the pancreas. Their relative density decreases from the head to the tail, with the head being the highest. Differently, in the pancreatic parenchyma, the postganglionic sympathetic nerve fibers are evenly distributed (17). In addition, nerve fibers capable of releasing calcitonin gene related peptide (CGRP) and substance P (SP) are distributed in the exocrine tissue of the pancreas and the islets (18).

## 3 Pain associated with PNI in PDAC

**Figure 1:**
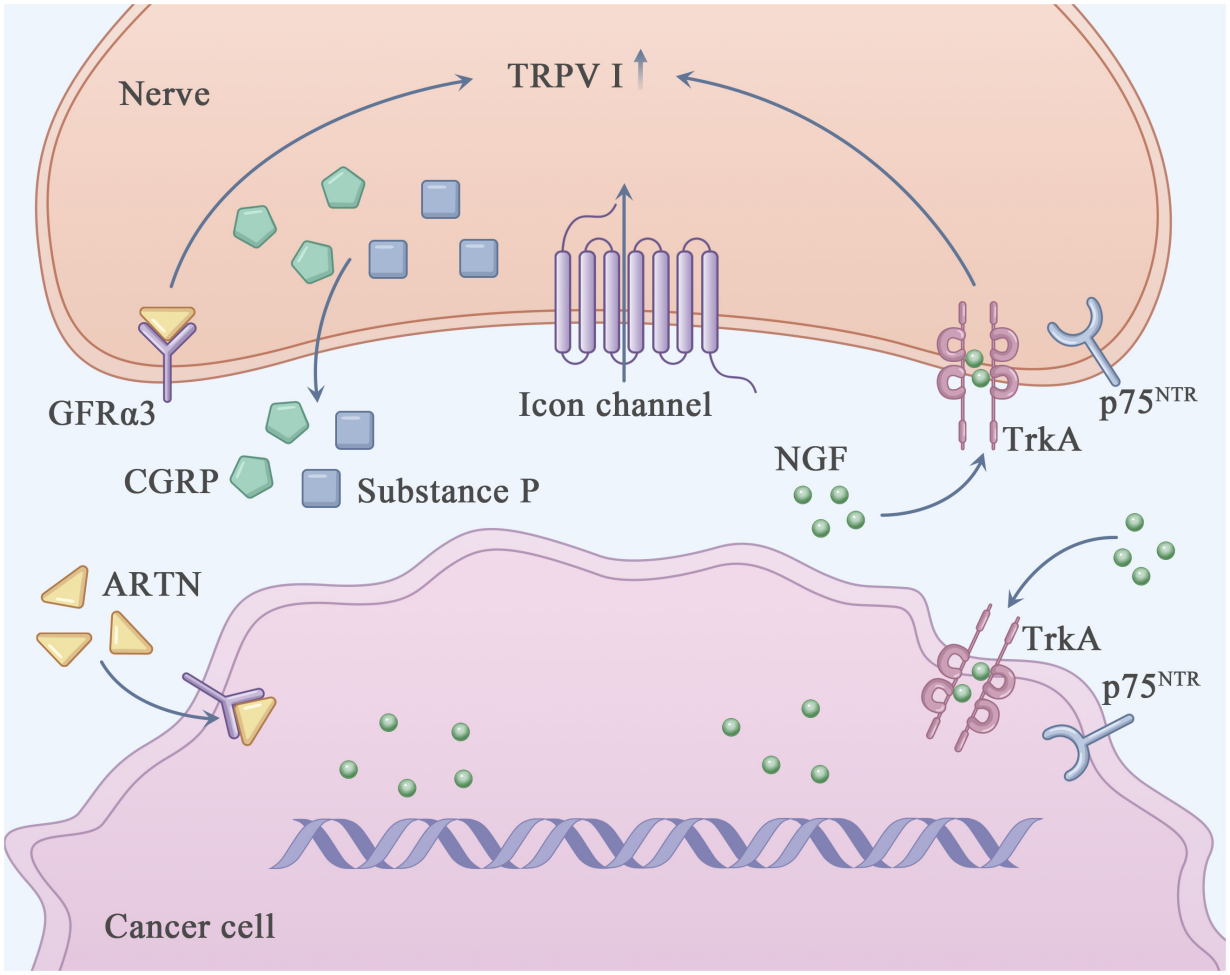
Perineural invasion related pain in PDAC. This figure shows the known major molecules in the pain generation process of PDAC. Nerve growth factor (NGF) interacts with TrkA and/or p75^NTR^ through autocrine and paracrine ways to directly activate and sensitize sensory nerves closely related to pancreatic cancer. Transient receptor potential cationic channel V subfamily 1 (TRPV1) acted as an ion channel, causing neurons to release pain related neurotransmitters such as Calcitonin-related peptide (CGRP) and substance P, which in turn emit pain signals through the central nervous system. The increased expression of Artemin (ARTN) increased the expression of TRPV1.

The clinical symptoms of PDAC mainly include abdominal pain, emaciation, diarrhea, jaundice and even bleeding (19). In addition, most patients with pancreatic cancer experience abdominal or back pain. Pancreatic cancer-related pain occurs through various mechanisms involving physical and chemical factors (20). From the physical aspect, pancreatic cancer cells invade the nerve plexus and destroy the nerve sheath, increasing the susceptibility of nerves to harmful stimulation by the ECM and eventually leading to pain. The positive feedback loop between pancreatic cancer cells and nerves may accelerate this process (21). In addition, invasion of the perineural space may promote the proliferation of pancreatic cancer cells in the limited space, resulting in pain (22). From the chemical aspect, various cytokines and growth factors derived from pancreatic cancer cells and nerves are involved in PNI-related pain (**Figure 1**).

Among the molecular factors involved in PNI, nerve-expressed transient receptor potential cationic channel V subfamily 1 (TRPV1) and Nerve growth factor (NGF) have been intensively investigated in studies on PDAC (23).

NGF is secreted by pancreatic cancer cells and TME-resident stromal cells, including tumor-associated immune cells and cancer-associated fibroblasts (24). It can directly bind to and activate sensory nerves expressing TrkA, p75^NTR^ and TRPV1, resulting in neurogenic inflammation and an elevated pain response (25, 26). TRPV1 (27), a non-selective cation channel (with a preference for sodium and calcium ions), is expressed in the central nervous system and sensory ganglia and participates in the regulation of pain response (28). It can be activated by various physical (thermal and mechanical stimulation) and chemical (capsaicin [an ingredient of chilli] and neurotrophic factors such as NGF (29), GDNF (30) and ARTN) factors (31, 32). Activation of TRPV1 may result in the depolarization of neurons and release of pain-related neurotransmitters, such as calcitonin-related peptide (CGRP) and substance P, which transport pain signals to the central nervous system (33). Li et al. reported that overexpression of ARTN in pancreatic cancer cells and Schwann cells increased the expression of TRPV1, thereby triggering capsaicin-related pain (34). The formation of the ARTN–GFRα3–TRPV1 complex in neurons may enhance TRPV1 function and pain response. Qin et al. reported that honokiol (HNK), a natural compound extracted from *Magnolia* spp, efficiently downregulated TRPV1 and relieved PNI-related pain in a mouse model of PDAC (35, 36). In addition, PNI-related pain can be alleviated by inhibiting CCL2–CXCR10. Although the L1-CAM–p38MAPK pathway has been reported to be involved in PNI-related pain in PDAC, the precise underlying mechanism warrants further investigation (25).

As the accompany process of vascularization new nerve fibers invasion during tumor progression, primary experiment data revealed the closely relationship between pain with the vascular endothelial cell derived molecules, such as vascular endothelial growth factor (VEGF) (37), ARTN (38), interleukin-1 (39), epinephrine B2 (40) and prostaglandin (41). Therefore, we speculate that tumor neovascularization, which promotes the growth of new capillaries and nerve fibers, may be involved in PNI-related pain in PDAC.

## 4 Process of PNI in PDAC

**Figure 2:**
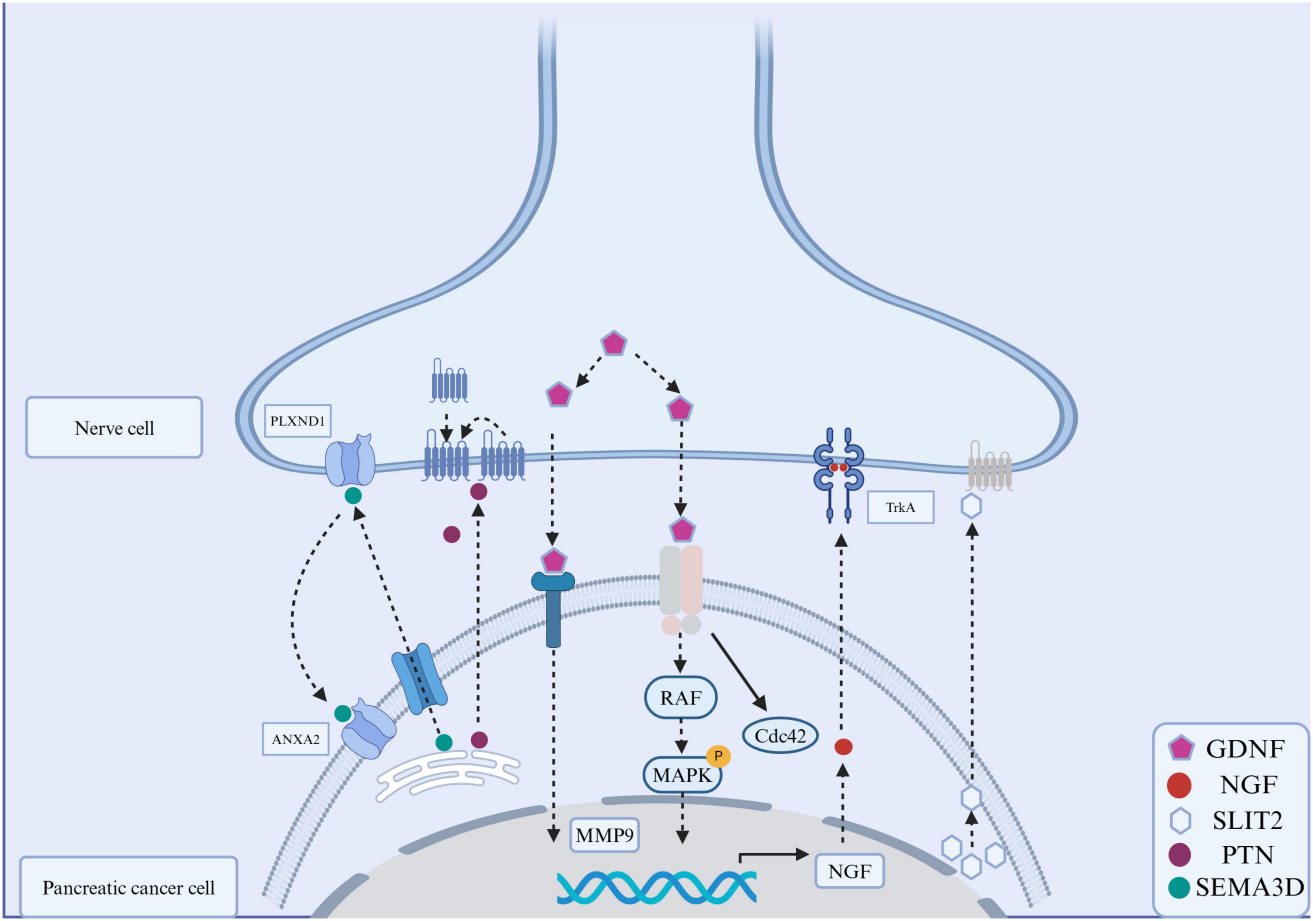
Signaling molecules in the perineural invasion of pancreatic cancer. The interactions between molecules expressed on cancer cells and nerve cells play an important role in PNI. These signaling molecules include secreted neurotrophins (such as nerve growth factor (NGF), which binds to tropomyosin-receptor kinase A (TRKA). Glial cell line-derived neurotrophic factor (GDNF) family: GDNF binds to RET, and downstream is RAF-MAPK. Annexin A2 (ANXA2) in nerve cells regulates the secretion of axonal SEMA3D, which binds to and activates PLXND1. The interaction between slit-guiding ligand 2 (SLIT2) and its receptor, roundabout guidance receptor 1(ROBO1), enhanced the motility and invasiveness of PDAC cells.

**Figure 3:**
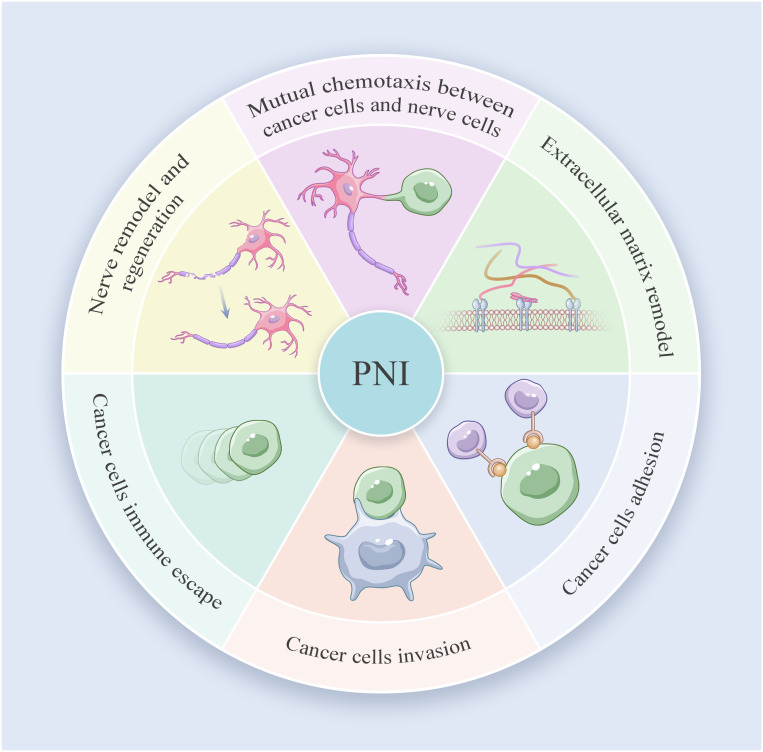
Perineural invasion journey in PDAC.

PNI involves a close and complex interaction among cancer cells, nerve cells and TME, which results in the dissemination of cancer cells within the perineural space. The dynamic communication between nerve and PDAC cells further facilitates the immune escape, growth and metastasis of tumors. Ayala and her colleagues found that DU-145 cells co cultured with neurons showed high expression of axon guiding molecule signaling protein 4F (S4F), and increased S4F induced neuronal axon length increase (42). A decrease in S4F expression led to a decrease in axonal growth. Proved that cancer cells could drive the generation of nerve axons (42). In addition, the removal of parasympathetic and sympathetic nerve innervation resulted in tumor inhibition in mice. The gene deletion of β2- and β3-adrenergic receptors in stromal cells could also inhibit early tumor progression (43). On the contrary, the stimulation generated by the parasympathetic nervous system contributes to the progression, invasion, and metastasis of tumors in the later stage. Indicating that cancer-related neurogenesis could also drive tumor progression. Amit et al. and Chen et al. firmly believed that PNI was mainly triggered through a closed-loop system, involving direct communication between nerve cells and the tumor microenvironment, improving tumor survival rate, avoiding cancer cell apoptosis and neuronal adhesion (44, 45). Based on previous studies and reviews, the process of PNI in PDAC can be divided into the following six steps: mutual chemotaxis between cancer and nerve cells, ECM remodeling, cancer cell adhesion and invasion, immune evasion of cancer cells and nerve remodeling and regeneration (**Figure 2**). Identifying specific and sensitive regulators of PNI and understanding the molecular mechanisms underlying PNI may guide the development of efficient diagnostic and therapeutic strategies (**Figure 3**).

### 4.1 Chemotaxis of cancer and nerve cells

**Figure 4:**
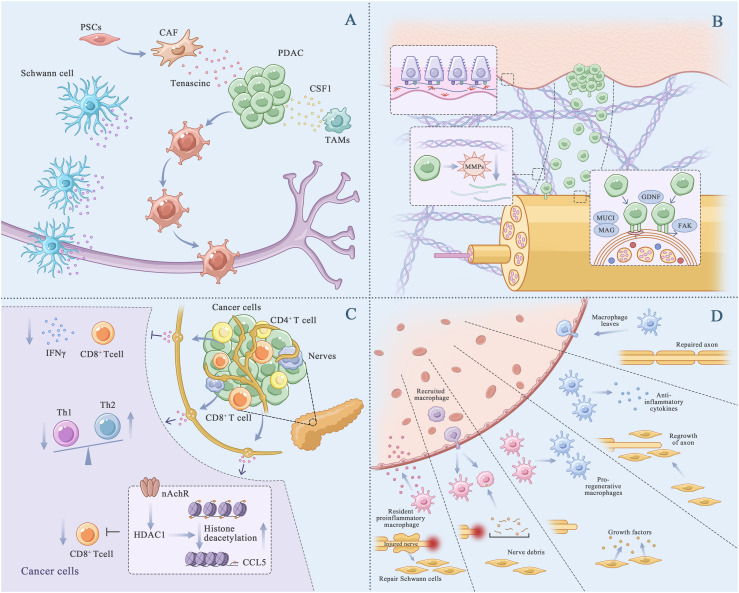
PDAC PNI progression. **(A)**, Cancer cells and nerve cells chemotaxis. The interactions between molecules that are expressed on the cancer cells and the peripheral nerves have an important role in PNI. TAMs and PSCs promoted the chemotaxis. TAMs (Tumor associated macrophages). PSCs (Pancreatic stellate cells). **(B)**, Cancer cells remodel extracellular matrix, adhesive and invade nerve cells. Cancer cells downregulated epithelial characteristics, and activate mesenchymal characteristics. Experienced EMT cancer cells released matrix metalloproteinase (MMPs), degraded and broken through the basement membrane, remodel the ECM and form a low-resistance corridor between the cancer cells and nerve cells. MUC1 promotes cancer cell invasion by activating β-catenin and inducing epithelial-mesenchymal transformation. **(C)**, Immune evasion of cancer cells. The increase of acetylcholine in the pancreatic vagus nerve downregulated the expression of CCL5 in cancer cells, inhibits the aggregation of CD8+T cells, and inactivated CD8+T cells, promoting cancer cell survival. **(D)**, Nerve remodeling and regeneration. The Schwann cells recruited after nerve injury arrived at the damaged site for repair. In this process, macrophages release anti-inflammatory substances to help with axonal repair.

In PDAC, PNI results from the coordinated activity of both cancer and nerve cells. Both cell types release various factors that promote their mutual chemotaxis (46). In the following sections, we discuss the precise roles of cancer and nerve cells in PNI (**Figure 4A**).

#### 4.1.1 Role of cancer cell-derived factors in the initiation of PNI

Cancer cells secrete various growth factors (NGF and hepatocyte growth factor [HGF]), chemokines and metabolites in an autocrine and/or paracrine manner. These molecules are involved in the initiation of PNI in PDAC. Upregulated NGF can bind to its receptors TrkA and p75^NTR^ on pancreatic cancer cells and nerve cells (47), facilitating cancer cell migration and invasion as well as nerve cell survival, growth and development (48). The interaction of HGF family members with the proto-oncogene receptor c-Met promotes PNI either directly by activating mTOR/NGF or indirectly by enhancing NGF secretion. Inhibition of c-Met suppresses the migration of cancer cells along the nerves, attenuates the damage caused by cancer cells to the sciatic nerve and protects the function of the sciatic nerve *in vivo (* 49, 50).

Cancer cell-derived C-X-C-based chemokine ligand (CXCL) and its related receptor (CXCR) on nerve cells can facilitate PNI in PDAC. The interaction of CX3CL1 with CX3CR1 promotes the migration of PDAC cells (MiaPACA2, T3M4, PANC1 and ASPC1 cells) to nerve cells (51). In addition, Nodal, which plays a key role in embryonic neural development, can promote the initiation of PNI in PDAC. Overexpression of Nodal in pancreatic cells upregulates the expression of NGF, BDNF and GDNF, enhancing the migratory and invasive abilities of cancer cells. A study showed that Nodal promoted the nerve sphere-forming ability in a 3D nerve invasion model involving the coculture of nerve and pancreatic cancer cells. Therefore, inhibiting Nodal expression may represent a promising strategy for targeting PNI in the treatment of pancreatic cancer (52).

#### 4.1.2 Role of nerve cell-derived factors in the acceleration of PNI

Nerve cells secrete growth factors (NGF and GDNF), chemokines and metabolites in a paracrine manner. These molecules have been reported to be involved in PNI in PDAC. GDNF accelerates the migration of *Kras*-mutated pancreatic cancer cells to nerve cells through the RET–phosphatidylinositol 4,5-diphosphate-3-kinase catalytic pathway. Inhibiting the release of GDNF from nerve cells has been shown to prevent PNI in a mouse model of PDAC (53). Soluble GFRα1 secreted by nerve cells may enhance PNI (54). Chernichenko et al. used siRNA library screening and found that the GDNF–RET–βPix–Cdc42 pathway guided the migration of pancreatic cancer cells to nerve cells. This pathway may serve as a promising therapeutic target for PDAC (55). Artemin, a GDNF family member, can initiate PNI through the GFRα3–NF-κB/CXCR4 signaling pathway (56). CXCL12 secreted by DRG in a paracrine manner can attract PDAC cells to the periphery of DRG (57). Norepinephrine activates adrenaline receptor β2 (ADRβ2), cAMP-dependent protein kinase and activating transcription factor 3 to induce the chemotaxis of cancer cells toward nerves (58). Glutamate is an important excitatory neurotransmitter in the nervous system, accounting for 60% -70% of the total number of synapses. Glutamate has two types of receptors: ion affinity receptors (NMDA) and metabolic receptors (59). The downstream signals of NMDA, MEK-MAPK and CaMK, induce tumor invasion in a mouse model of pancreatic neuroendocrine tumors (60). A recent study by Li et al. showed that glutamate in nerve cells can induce calcium influx into PDAC cells through N-methyl-D-aspartate receptor (NMDAR), thereby activating the downstream calcium-dependent protein kinase CaMKII/ERK–MAPK pathway and promoting the transcription of METTL3. Subsequently, METTL3 upregulates the mRNA expression of hexokinase 2 (HK2) through N6-methyladenosine (m6A) modification, thereby promoting glycolysis and PNI in PDAC (61). The interaction between Schwann cells and cancer cells can be regulated at multiple levels. Schwann cells can communicate with cancer cells by secreting proteins, including L1 cell adhesion molecule (L1-CAM) (62) and TGFβ (63). In a study, immunohistochemical analysis of PDAC tissue samples showed that L1-CAM was highly expressed in cancer cells and adjacent Schwann cells of the affected nerves. L1-CAM secreted by Schwann cells affected the chemotaxis of cancer cells by activating the MAP kinase signaling pathway (62).

Stromal cells present in TME and factors secreted by them are involved in the chemotaxis of cancer and nerve cells. Pancreatic stellate cells (PSCs), which account for 50% of all stromal cells in the PDAC microenvironment, release the ECM glycoprotein tenascin C, which enhances the chemotaxis of cancer and nerve cells (64). Aberrant activation of hedgehog signaling is involved in the communication among tumor cells, PSCs and nerve cells (65). Pancreatic cancer cells release hedgehog signaling molecules to activate the hedgehog pathway in PSCs, facilitating the chemotaxis of cancer cells toward nerve cells. Tumor-associated macrophages (TAMs) result in the formation of an immunosuppressive TME in PDAC. Studies have shown that TAMs contribute to a poor prognosis in PDAC with PNI (66). Colony-stimulating factor 1 (CSF1) secreted by PDAC cells can recruit and stimulate macrophages to promote the chemotaxis of cancer cells (67).

Not all neurotrophins are highly expressed in PDAC cells. For example, Göhrig A et al. showed that the mRNA expression of slit-guiding ligand 2 (SLIT2), a neurotrophic protein related to cell migration, was low or absent in PDAC cell lines and tumor tissues from patients with PDAC (68). Functional studies have shown that overexpression of SLIT2 in human PDAC cell lines (MiaPACA2 and PANC1) suppresses cell migration and invasion (68). Conversely, blocking the interaction between SLIT2 and its receptor roundabout guidance receptor 1 (ROBO1) enhances the motility and invasiveness of PDAC cells, further supporting the inhibitory effects of SLIT2 on cancer cell migration and invasion (68).

### 4.2 Extracellular matrix remodeling

Under the chemotactic action of nerve cells, cancer cells initially undergo EMT and subsequently remodel the ECM. During EMT, cancer cells lose epithelial characteristics and acquire mesenchymal characteristics. ECM is a polymeric network of proteins, glycoproteins, proteoglycans and glycosaminoglycans that supports and compartmentalizes tissues while regulating cell fate and function (69). It is roughly divided into two parts, namely, the interstitial connective tissue matrix and the basement membrane (BM). After undergoing EMT, cancer cells release matrix metalloproteinases (MMPs), degrade and penetrate the BM (70–72) and remodel the ECM to create a low-resistance corridor between cancer and nerve cells, thereby crossing the ECM (**Figure 4B**).

BM is composed of an interrelated network of type IV collagen, laminins, nidogen and sulphated proteoglycans that lies beneath epithelial and endothelial cells and surrounds muscles, nerves, adipocytes and smooth muscle cells (73). Molecules secreted by cancer and stromal cells participate in the penetration of BM. Proteolysis of MMPs disrupts BM, which is necessary for pancreatic cancer cells to pass through the barrier. MMP2 and MMP9 lead to polysaccharide deficiency and interrupt the activity of extracellular fibronectin and type IV collagen, which play an important role in the penetration of BM by cancer cells (74). GDNF activates MMP9 by binding to the RET tyrosine kinase receptor and activating two downstream signaling pathways: PI3K–AKT and RAS/RAF–MEK-1–ERK1/2 pathways, which are essential for the activation and expression of MMP9 (53, 75). Stromal cells secrete various factors that indirectly regulate the expression and function of MMPs. Schwann cells surrounding nerves can release L1-CAM, activate downstream STAT3, upregulate the expression of MMP2 and MMP9 and promote the degradation of ECM (62). The expression of MMPs is remarkably high in cocultured PSCs and PDAC cells (76). Upregulated galactose agglutinin 1 (LGALS1) in stellate cells activates SRC signaling in PDAC cells, leading to the transcription of MMPs, which is necessary for the degradation of ECM and the passage of cancer cells through BM (71). *In vitro* studies have shown that activated macrophages can stimulate human PDAC cells (PANC1 and MiaPACA2) to secrete MMP1 (77). Soluble MMP1 induces DRG to release substance P (SP); activates killer cell lectin-like receptor B1 (KLRB1) and consequently leads to ECM degradation through the SP, KLRB1 and MAPK pathways (77).

In an *in vivo* setting, ECM degradation involves not only the disruption and penetration of BM by cancer cells but also the formation of a low-resistance corridor in ECM to facilitate cancer cell migration. Cancer cells follow a certain concentration gradient, and aggregation results in mechanical pressure (78). PDAC cells migrate toward never cells along the GDNF concentration gradient. Cancer-associated fibroblasts (CAFs) are highly differentiated stromal cells that promote ECM remodeling in tumors (79). CAF-derived TGFβ stimulates the synthesis of collagen in neighboring fibroblasts, thereby increasing the stiffness of the tissue. The resulting mechanical stress in the connective tissue leads to the collapse of neighboring structures. Subsequently, the dense structure formed through fibrosis surrounding a group of PDAC cells increases the internal pressure and leads to the rupture of glandular structures (69). The formation of high-density fibers helps to track the movement of cancer cells.

### 4.3 Cancer cell adhesion

After they migrate to nerve cells, cancer cells adhere to the neurilemma by binding to cell adhesion molecules and their related receptors (**Figure 4B**). Overexpressed Mucin 1 (MUC1), a type I transmembrane protein, on pancreatic cancer cells can selectively bind to MAG on peripheral nerve cells through the abnormal glycosylation extracellular domain, contributing to selective adhesion between the two types of cells (80). In addition, overexpressed MUC1 promotes cancer cell invasion by activating β-catenin and inducing EMT. GDNF treated pancreatic cancer cell line displayed silk foot and tablet lipid formation enhanced extracellular matrix proteins adhesion ability, which played an important role in the PDAC-related PNI (81). In a study, high-throughput genetic analysis of a neurotrophin sorting model showed that kinesin family member 14 (KIF14) and Rho GDP dissociation inhibitor β (ARHGDI β) were involved in the recruitment of adhesion molecules to the cell membrane, however, the exact underlying mechanism warrants further investigation (82). The interaction between CXCR1 and CX3CL1 facilitates the co-localization of β1 integrin and focal adhesion kinase (FAK) to stabilize adhesion between nerve and cancer cells (51). Nerve cell adhesion molecule 1 (NCAM1) expressed on Schwann cells can enhance cell–cell adhesion through cadherins; however, modification of NCAM1 by polysialic acid counteracts these effects (83, 84).

### 4.4 Cancer cell invasion

The nerve bundle membrane, which acts as a protective barrier, contains laminin, fibronectin and type IV collagen. After cancer cells infiltrate the nerve bundle membrane, cancer cells and neighboring cells secrete numerous factors that promote the proliferation and migration of cancer cells along the nerve fibers (**Figure 4B**) (85).

Damaged nerve fibers produce abundant neurotrophic hormones and attract more cancer cells, creating a vicious cycle. PAP/REG3A (PAP/REG3β), a type C lectin-like secretory protein, can promote the growth of cancer cells in the nerve space, resulting in an increase in mechanical pressure in nerve fibers (86, 87). Upregulated SDC3 in nerve cells may lead to the aggregation of PTN^+^ PDAC cells around nerve cells, thereby promoting cancer cell invasion (88, 89). In the nerve space, cancer cells require more nutrients or energy to support their growth and metastasis by altering their metabolism. Axons and dorsal root ganglia (DRG) secrete serine to provide additional energy to cancer cells. In the case of serine deprivation, PDAC cells express and secrete more NGF by upregulating its translation, thereby enhancing the movement of axons toward the tumor nest.

In addition to the direct communication between cancer cells and nerve fibers, stomal cells, such as CAFs, participate in and enhance cancer cell invasion. In PDAC, CCL2 secreted by Schwann cells can recruit CCR2 monocytes, which further differentiate into macrophages, release protease B and contribute to the degradation of nerve bundle membrane proteins (90). TGFβ produced and released by Schwann cells can enhance the invasive ability of PDAC cells. Ferdoushi et al. showed through proteomic analysis that TGFβ (TGFβ-1, TGFβ-2, TGFβ-BI) played an important role in Schwann cells stimulating pancreatic cancer cell migration, invasion and metastasis, but the specific functional analysis is unknown (91). Roger et al. also reported that TGFβ rich in Schwann cell culture medium could promote the migration and invasion of Capan-2 cells (63). In addition, tumor-activated Schwann cell trajectories (TASTs) can promote the migratory and invasive abilities of PDAC cells. Non-myelinated Schwann cells form TASTs, resulting in a poor prognosis in PDAC. In TASTs, dynamic SCs form trajectories as cancer-related pathways and enhance the migratory ability of cancer cells (92). Activation of CAF-derived MMP2 by membrane type I matrix metalloproteinases (MT1-MMPs) contributes to the degradation of nerve bundle collagen and facilitates the diffusion of cancer cells in the perineural space (93, 94).

Abnormalities in mitochondrial function and glucose metabolism have been associated with cancer cell invasion, as these metabolic changes may be related to the aetiology of neurodegeneration and nerve injury. Oxidative stress can induce chronic neuroinflammation and the transformation of astrocytes from the neurotrophic to neurotoxic phenotype, which may inhibit nerve cell proliferation. In addition, the binding of NGF to TrkA receptors can induce the Warburg effect in PDAC and DRG cells. Goetze et al. showed that lactate can promote cancer cell invasion (95). The nervous system contains abundant lactate, which is produced by Schwann cells through aerobic glycolysis to support the propagation of action potentials along axons (96).

### 4.5 Immune evasion

After invasion, cancer cells attempt to evade elimination by neighboring immune cells. Neurotransmitters regulate anti-immune responses. In particular, sympathetic and parasympathetic nerve fibers release norepinephrine (NE) and acetylcholine (ACh) in tumors to reduce the anti-tumor immune response (**Figure 4C**) (97, 98). Catecholamine triggers immunosuppression in the TME of lung cancer, leading to the re-polarization of M1 macrophages to the M2 phenotype and the aggregation of M2-polarized macrophages and MDSCs. In addition, it reduces the proportion of anti-tumor dendritic cells (DCs), resulting in the synthesis and release of IL-10 and VEGF, inhibition of immune responses and promotion of angiogenesis (99). The impact of adrenergic stress and subsequent catecholamine release on T lymphocytes in the TME was also studied in mouse models of colon cancer and melanoma (100, 101). However, role of catecholamine in PNI in PDAC remains unclear. Catecholamines (adrenaline, NE, DE), also known as stress mediators, have been recognized for their role in cognitive, emotional changes, stress-related diseases, cancer occurrence, and tumor metastasis (102). Based on the above reports, we assumed that immune checkpoint blockade in PDAC is driven by adrenergic stress, leading to increased expression of certain immune checkpoint molecules such as PD-1, FOXP3, and LAG3, thereby triggering T cell depletion.

Macrophages typically had a high content in the TME immune cell population and mainly exerting immunosuppressive effects and participating in multiple stages of PDAC PNI progression (4, 9, 103). TAMs were mainly differentiated from inflammatory monocytes. As mentioned above, myeloid progenitor cells in bone marrow differentiated into inflammatory monocytes under the mobilization of the CCL2/CCR2 axis (104). In the progress of PDAC PNI, inflammatory monocytes at the injured site differentiated into TAMs (105), which participated in the inhibition of various immune responses. Played an important role in dealing with nerve injury and various environmental disturbances (106). TAMs could be divided into M1 and M2 subtypes (106). Type M1 usually played an anti-tumor function, while type M2 played an immunosuppressive function (103). TAMs were usually considered M2 type and secreted various cytokines such as CCL5 and TNF to enhance PNI (107). In the PDAC PNI progression, nuclear factor kappaB (NFκB) could promote the repolarization of macrophages from M1 to M2 and exert the immunosuppressive function of macrophages (108, 109). In addition, OC et al. reported that endoneurial macrophages could be transformed into microglia/macrophage subsets, which could promote nerve regeneration and anti-tumor (67). Endoneurial macrophages were recruited to the front of the tumor by colony stimulating factor-1 secreted by tumors. Activated endoneurial macrophages secreted high levels of GDNF, promote RET phosphorylation on PDAC cell membrane, activated downstream MAPK and PI3K pathways in cancer cells, and enhance PNI (67).

Cancer-associated fibroblasts (CAF) were highly differentiated stromal cells that supported tumor invasion by stimulating angiogenesis, cancer cell proliferation and matrix remodeling (110). In PDAC, CAF was thought to differentiate from pancreatic stellate cells (111). CAF has been proved to be the source of paracrine growth factors, which affected the growth of cancer cells through paracrine, so that some cancer cells could survive (112). For example, M.J et al. reported that TGFβ from CAF induced fibroblasts to react and synthesize collagen, which hardened the surrounding tissue, and the resulting mechanical stress destroyed nearby blood vessels, leading to hypoxia (113). At this time, Schwann cells were activated by interleukin-6 (IL-6) produced by cancer cells, facilitated PNI, which was accompanied by changes in hypoxia-related signaling pathways, such as HIF1α (114). In addition, the interruption of glandular structure is induced by the internal high pressure of CAF fibrosis. At the same time, the changes of fiber structure also make it easier for cancer cells to spread and metastasize (112). CAF and cancer cells work together in PDAC PNI: CAF makes it easier for cancer cells to invade, while cancer cells polarize to promote the spread of CAF. CAF could also promote cancer invasion by secreting matrix remodeling factors (115, 116). A.F et al. and V.S et al. showed that in the process of PNI, CAF at the injured site produced inactivated MMP2, which is then activated by membrane type I matrix metalloproteinases (MT1-MMPs) from cancer cells, then could degrade extracellular collagen of nerve membrane and promote the process of PDAC PNI (94, 117).

T cells, one of the main components of the immune system, originated from the thymus and perform the function of killing infected host cells or cancer cells (118). As mentioned earlier, in PDAC PNI, the vagus nerve played an immunomodulatory role by controlling the infiltration or activation of T cells (119). ACh released from vagus nerve inhibited the expression of CCL5 in PDAC by mediating histone deacetylation. Low levels of CCL5 damage the recruitment of CD8+T cells, leading to immunosuppressive tumor microenvironment. Interferon-γ from CD8+T cells was also inhibited by ACh, which promoted the differentiation of T cell helper 2 cells and decreased T cell helper 1 cells (119). And vagotomy destroyed the PNI process and inhibited tumor growth *in vivo (* 119). However, BW et al. reported that subseptal vagotomy could accelerate the tumor formation of Kras^G12D^-driven PDAC in mice (120). These two distinct results suggest that cholinergic nervous system plays a dual role in PDAC and participates in the recruitment and activation of T cells.

### 4.6 Nerve remodeling and regeneration

Cancer cells, with the help of nerve cells, evade the immune system, continue to proliferate, metastasize along nerve fibers and infiltrate them. The damaged nerve fibers are repaired and regenerated through the combined action of Schwann cells, macrophages and CAFs and continue to exert chemotactic effects on cancer cells, forming a vicious cycle (**Figure 4D**). Once damaged and invaded by cancer cells, axons may trigger the dedifferentiation and activation of Schwann cells through the release of neuromodulators (NRGs) from remaining neurons (121). In PDAC, overexpression of Artemin, a member of the α ligand family, promotes neuronal remodeling and nerve fiber proliferation around tumors through Ret/GFRα3 receptors (122). Damaged Schwann cells play an important role in maintaining neuron survival by producing various neurotrophic factors and cell surface proteins, releasing pro-inflammatory mediators to alter the local signaling environment, participating in axonal maintenance and post-injury repair and contributing to subsequent nerve regeneration (123). After partial peripheral nerve injury, the continuous demyelination of peripheral nerves relieves the inhibition of axon growth and promotes nerve germination. CAFs contribute to axonal growth by secreting Eph-B, which activates the EphB2 receptor on Schwann cells, and inducing the migration of Schwann cells within the nerve bridge through the Eph-B/EphB2 signaling pathway, thereby guiding axons to reach the injured site (124). As members of the membrane-associated or secretory glycoprotein family, signaling proteins are involved in axon guidance and cell migration, with axon guidance being the most important function. Annexin A2 (ANXA2) in nerve cells regulates the secretion of axonal SEMA3D (125), which binds to and activates PLXND1 (126), thereby promoting PNI in PDAC (127). The levels of interleukin-6 (IL-6), which is produced and secreted by CAFs and mast cells, are high in serum or tissue samples from patients with PDAC (128). After binding to corresponding receptors on Schwann cells, IL-6 activates the STAT3 pathway, triggering the growth of Schwann cells and promoting neuronal plasticity (128).

In addition, Mauffrey et al. demonstrated that neural precursor cells expressing the neural stem cell marker Doublecortin (DCX+) migrated from the region of neural origin to the niche of the tumor and differentiated into mature noradrenergic neuronal phenotypes (129). More DCX+cells were observed in high-risk cancer samples compared to low-risk samples. The depletion of DCX+cells also reduced the incidence of tumor lesions, and with the addition of DCX+neural precursor cells, tumor growth accelerated (129). Cancer stem cells could also form new neurons. Lu et al. found that cancer stem cells from gastrointestinal patients could differentiate into sympathetic neurons that produced tyrosine hydroxylase (TH) and parasympathetic neurons that produced vesicular acetylcholine transporters (130). On the contrary, these neurons could communicate with cancer cells in xenografts to promote tumor growth (130). In summary, cancer cells could promote the regeneration of nerve cells to a certain extent.

## 5 Treatment of PDAC with PNI

Surgery is the first-line treatment strategy for early-stage PDAC. PNI is detected in the early postoperative stages in most patients (131). The expanded resection scope includes the entire pancreatic structure, dissection of lymph nodes and removal of all relevant blood vessels (132, 133). Meanwhile, explore new methods for intraoperative use of staining techniques to identify neural structures and alleviate PDAC PNI (133). A combination of gemcitabine and Nab-paclitaxel or mFOLFIRINOX is commonly used to treat advanced PDAC with local or distant metastasis (134). Denervation of the pancreas using an ethanol-induced celiac plexus block represents a useful strategy for prolonging the survival of patients with advanced PDAC. A study showed that patients treated with ethanol-induced splanchnicectomy, a procedure for the surgical resection of splanchnic nerves, survived longer than patients in the control group (median survival, 9.15 vs. 6.75 months) (135). The frequency of PNI ranges from 70.8 to 93.0% in patients with PDAC treated with pancreatectomy and from 43 to 58% in patients treated with neoadjuvant therapy and pancreatectomy (9).

SBRT radiotherapy, a neoadjuvant treatment modality, is widely used for treating locally advanced pancreatic cancer; however, the overall radiation dose was not high, and does not reach the radiotherapy mode of high dose and low frequency (136). In a study, the incidence of PNI in patients with PDAC who received neoadjuvant radiotherapy (58%) was lower than that in patients who did not receive neoadjuvant radiotherapy (80%) (P = 0.002) (137). Preclinical studies have shown that radiotherapy may help limit PNI. In particular, 4Gy radiation can significantly reduce the release of GDNF in DRG, thereby inhibiting the invasive ability of MiaPACA2 cells (138). 8Gy radiation can lead to a decrease in the secretion of GDNF by the sciatic nerve, inhibiting the progression of PNI and protecting the sciatic nerve against cancer cell-induced damage *in vivo*. In addition, sustained low-dose irradiation with iodine-125 seeds has been shown to inhibit tumor growth and PNI in preclinical models, suggesting that local implantation of iodine-125 seeds in tumors can alleviate PNI-related pain in PDAC (139).

The characteristics of PDAC, a malignant tumor, were chemotherapy resistance and radiation resistance. 80% patients receiving surgical treatment experience recurrence and PNI, and over 50% patients receiving neoadjuvant therapy also experience recurrence and PNI (9, 137). PDAC PNI patients also have strong abdominal and back pain, and poor life quality (140). In this context, it is particularly important to promote research and development of new drugs targeting PDAC PNI.

At present, PNI targeted therapy has made certain progress, and several new drugs are undergoing various experiments in order to enter clinical practice. Such as anti-NGF drugs (141), anti-Trk receptor drugs (142), Resiniferatoxin (RTX) (26), oncolytic adenoviruses (143), and Honokiol (HNK) (36). In preclinical models of PDAC, the NGF-TrkA signaling pathway has enormous potential for drug development (25). Specific antibodies or genetic interference could be used to block NGF and alleviate pain, or specific tyrosine kinase receptor blockers could be used to delay tumor progression. The current anti-NGF antibodies, such as muMab911, Tanezumab, MNAC13, PHA-848125, and ARRY-470, all act by blocking the binding of NGF to its receptors. Tanezumab has already been tested in Phase III trials (144). In addition, in environments lacking serine/glycine, Trk-NGF inhibitors (LOXO-101) could reduce nerve innervation and slow down PDAC progression (142). Some data confirm that Resiniferatoxin (RTX) could promote apoptosis of pancreatic cancer cells and reduce pain awareness, which means that RTX may become a new effective drug for PDAC patients to fight against neurogenic pain (26). The model of PNI demonstrated that attenuated herpes simplex virus could detect invading nerves through fluorescence imaging during surgery, making lesion clearance more thorough *in vivo (* 145). T. K et al. reported that oncolytic adenoviruses (OBP-301 and OBP-702) could inhibit the migration and invasion of pancreatic cancer cells (143). OBP-702 acted on ERL signaling, blocking the migration and invasion of PDAC cells. Of course, further research is needed to explore the safety of OBP-301 and OBP-702 in PDAC patients (143). Magnolol (HNK) was a polyphenolic compound extracted from plants of the Magnolia genus. HNK could alleviate PNI. In mechanism, HNK may inhibited PDAC PNI by inhibiting the activation of Smad2/3, leading to the reduction of NGF and BDNF in pancreatic cancer cells (36). These promising studies may provide effective methods for treating PDAC PNI in the future, reducing patient pain and prolonging patient survival.

## 6 Conclusion and prospect

PNI is a complex phenomenon that involves multidirectional communication among nerve cells, tumor cells and TME. Numerous studies have investigated the pathological processes and mechanisms involved in the development of PNI in PDAC. Targeting these processes or mechanisms represents a promising therapeutic strategy for PDAC. The first half of this review starts with the pain caused by PNI in pancreatic cancer, and combined with the function of nerves, describes the current research progress on nerve pain in pancreatic cancer. By understanding the mechanism of PNI and pain in pancreatic cancer, we may be able to find more therapeutic strategies to delay and block PNI. In the second half of this review, the PNI process of pancreatic cancer is described from six steps: mutual chemotaxis between cancer and nerve cells, extracellular matrix (ECM) remodeling, cancer cell adhesion and invasion, immune evasion of cancer cells and nerve remodeling and regeneration. The whole invasion process is the result of many factors. In this process, identifying specific molecular targets or exploring new ways to inhibit PNI may have potential implications for the treatment of PDAC.

In the past few decades, research on PDAC PNI has been focused on pathology and molecular biology. Due to methodological limitations, there is an urgent need for a new approach to help scholars further understand PNI. With the rapid development of machine learning and artificial intelligence, research on PNI seems to have found new directions. Artificial intelligence (AI) is an exciting new technology that can mimic human cognitive learning processes and automate specific tasks (146). The more data AI algorithms provided, the more reliable they are in executing tasks. According to reports, artificial intelligence has successfully identified PNI in prostate cancer tissue (147). They developed an artificial intelligence (AI) algorithm based on deep neural networks, which measured the PNI of 7406 patients, reducing the workload of pathologists and more clearly demonstrating the relationship between perineural invasion and poor prognosis (148, 149). Fortunately, Borsekofsky et al. developed a highly sensitive artificial intelligence algorithm in the field of PDAC PNI to detect PNI in PDAC surgical specimens. Compared with pathologists analyzing it separately, this algorithm significantly improved time, efficiency, and accuracy (146). From the current perspective, the main function of artificial intelligence is to assist pathologists in early diagnosis of PNI (150). AI in the field of pathology is still very young and will continue to mature in the future.

However, some issues deserve further study: (1) Existing techniques for screening specific molecular mechanisms have not been widely used in studies on PDAC with PNI. Moreover, large-scale molecular data on cancer and nerve cells involved in PNI are lacking. In recent years, single-cell sequencing has improved our understanding of biological systems. Given that the function of many biological systems depends on their spatial organization, novel techniques for analyzing single-cell spatial transcription and metabolism have emerged with the development of high-throughput technologies and the improvement of computational methods. Single-cell spatial transcriptomic techniques can be used to elucidate PNI in pancreatic cancer, understand its role at different spatial points and reveal the underlying mechanisms. With the development and progress of molecular biology and genomics, valuable molecular markers of PNI have been identified, which may provide important clinical evidence for the early prognosis and prompt treatment of patients with PDAC.

(2) Many clinical trials on PDAC lack preclinical modeling in either GEMMs or orthotopic PDX models, which are more representative of human diseases than commercial PDAC cell lines. Although preclinical studies have used commercial PDAC cell line-derived xenograft mouse models with encouraging *in vivo* results, clinical trials have been unable to proceed. In addition, as research on immunology and PNI is gradually progressing, it is important to use immunocompetent GEMMs (or humanized PDX models) to verify anti-tumor PNI phenotypes and mechanisms. In addition to models of tumor–nerve interactions, organic PDAC cultures derived from patient sources hold great promise in predicting clinical responses to treatment. However, the predictive ability must be validated in prospective clinical trials. Developing novel treatment methods for PDAC is challenging. In addition to the ongoing clinical trials, we have also noticed many clinical conversion failures that attempt to achieve therapeutic benefits by blocking various processes of PNI. Clinical trials should be designed based on strong preclinical data to optimize the chances of success. Future developments and discoveries heavily depend on limited data from experimental and animal models used to simulate PNI. Elucidating the precise mechanisms underlying PNI particularly relies on methodological advances, which is an important focus of future research. In addition, studies investigating the reciprocal interactions between the fields of immunology and metabolism and pipelines that incorporate mathematical modeling as well as artificial intelligence- and machine learning-based methods are required.

## Author contributions

YS: Software, Writing – original draft. WJ: Data curation, Validation, Writing – review & editing. XL: Methodology, Project administration, Writing – review & editing. DW: Conceptualization, Methodology, Writing – review & editing, Funding acquisition.
